# Neurochemical, structural and neurobehavioral evidence of neuronal protection by whey proteins in diabetic albino mice

**DOI:** 10.1186/s12993-015-0053-0

**Published:** 2015-02-13

**Authors:** Jamaan Ajarem, Ahmed A Allam, Hossam Ebaid, Saleh N Maodaa, Sanad M AL-Sobeai, Ahmed M Rady, Ali Metwalli, Naif G Altoom, Khaled Elfakki Ibrahim, Mohammad I Sabri

**Affiliations:** Zoology Department, College of Science, King Saud University, Riyadh, 11451 Saudi Arabia; Department of Zoology, Faculty of Science, Beni-suef University, Beni-Suef, Egypt; Department of Zoology, Faculty of Science, Menia University, Minya, Egypt; Shaqra University Sajir College of Arts & Science, Shaqra, Saudi Arabia; Department of Food Science, College of Agriculture and Food Science, King Saud University, Riyadh, Saudi Arabia; Department of Dairy, Faculty of Agriculture, El-Minia University, El-Minia, Egypt; Oregon Health & Science University, Portland, OR USA

**Keywords:** Whey protein, Diabetes, Oxidative stress

## Abstract

**Background:**

Diabetes Mellitus (DM) is associated with pathological changes in the central nervous system (CNS) and alterations in oxidative stress. The aim of this study was to determine whether dietary supplement with whey protein (WP) could improve neurobehavior, oxidative stress and neuronal structure in the CNS.

**Methods:**

Animals were distributed in three groups, a control group (N), a diabetic mellitus group (DM) and a DM group orally supplemented with WP (WP).

**Results:**

The DM group of animals receiving WP had reduced blood glucose, significantly decreased free radical Diphenyl-picrylhydrazyl (DPPH) and lower lipid peroxidation in brain tissue. The WP group of animals showed improvement in balancing, coordination and fore-limb strength, oxidative stress and neuronal structure.

**Conclusion:**

The results of this study show that dietary supplementation with WP reduced oxidative stress, protected CNS neurons and improved the neurobehavior of diabetic mice.

## Background

Diabetes Mellitus (DM) is regarded as a major global epidemic of the 21^st^century [1]. DM is a complex and heterogeneous metabolic disorder characterized by hyperglycemia in several organs of affected individuals [2]. DM is associated with decreased physical activities brain atrophy and lesions [3].

Diabetic encephalopathy is characterized by impaired cognitive functions and neurobehavior, neurochemical changes and neuronal damage caused by the increased intracellular glucose level. Previous studies assessed the effect of chronic hyperglycemia on the function of neuronal mitochondria in the brain, the major site of reactive free radical production in streptozotocin (STZ) diabetic rats [4]. DM is characterized by disturbance in oxidative stress that plays a central role in tissue damage. The expression of reactive oxygen species (ROS), nitric oxide and nitric oxide synthase were found to be increased in mitochondria, whereas glutathione (GSH), peroxidase activity and manganese superoxide dismutase were reduced in DM [5,6]. Also, GSH was reduced but glutathione disulfide (GSSG) was increased in the brain of STZ rats [4]. The overproduction of reactive species induced by enhanced glucose oxidation might overwhelm the antioxidant defenses, leading to cell damage. It has been reported that normalizing mitochondrial superoxide production blocks the pathways of hyperglycemic damage [7]. Recently, however, the unique role of brain mitochondrial dysfunction in experimental diabetes has been questioned and it has been suggested that extra-mitochondrial factors may be involved in the induction of oxidative stress in diabetes [8]. Emerging evidence showed that the increased oxidative stress and consequent oxidative damage in hyperglycemic conditions begins in the mitochondria, the major site of reactive oxygen species production [9].

The balance between oxidation and antioxidation and is critical for maintaining a healthy biological system. Hyper physiological burden of free radicals causes imbalance in the homeostasis between oxidants and antioxidants in the body. This imbalance leads to oxidative stress in aging and various human diseases like atherosclerosis, stroke, diabetes, cancer and neurodegenerative diseases such as Alzheimer disease and Parkinson disease [10]. The majority of oxidant species involved in physiological oxidative events are anion superoxide (O^−^_2_), hydroxyl radical (HO^−^), nitric oxide and peroxinitrite. In stress conditions, these species can initiate further deleterious effects on biomolecules and cause cellular damage. These effects can be lipid peroxidation [11], protein oxidation [12], DNA damage [13], oxidation of the reducing equivalents such as nicotinamides and thiols such as GSH and alterations in intracellular calcium homeostasis [14]. These phenomena can be prevented and/or reversed, by the cellular antioxidant capacity; GSSG may be converted to GSH through the enzymatic reaction catalyzed by GSH reductase and NADPH [15]. Recent accumulated evidence has shown that natural antioxidants can prevent and treat the onset of diseases caused and/or fostered by oxygen free radicals [16].

Diphenyl-picrylhydrazyl radical (DPPH) bleaching is one of the strategies has been used to evaluate the antioxidant properties of natural products; this method has shown to be rapid and simple that measures the capacity of herbal extracts and natural products to bleach the DPPH radical, a nitrogen-centered free radical [17]. In foods, antioxidants have been defined as substances that in small quantity are able to prevent or greatly retard the oxidation of materials such as fats [18]. However, biological antioxidants have a further broad definition, which includes systems such as metal transport proteins (e.g. transferrin, albumin, ferritin and ceruloplasmin) to prevent the redox properties of metal, antioxidant enzymes and factors involved in vascular homeostasis, signal transduction and gene expression [19]. Thus, the cellular antioxidant mechanisms involve suppressing of ROS formation, reducing oxygen free radicals (O^−^_2_, HO ^−^ , ROO^−^) and H2O2, sequestering metal ions, scavenging active free radicals, repairing and/or clearing the oxidative damage. The bioactivity of an antioxidant also depends on factors like its physico-chemical characteristics and in vivo radical generating conditions [20].

Proteins are essential for the maintenance and repair of body tissues. Camel whey protein (WP) is a heterogeneous group of proteins that include serum albumin, α lactalbumin, immunoglobulin, lactophorin and peptidoglycan recognition protein [21]. WP contains all of the essential and nonessential amino acids and is a good source of glutamine and the branched-chain amino acids that are necessary for cell growth [22]. WP has been found to significantly suppress hydroperoxide and ROS levels in liver and other tissues in mice by stimulating production of glutathione synthesis and cellular antioxidant defense [23]. Therefore, WP may be used as a therapeutic tool for oxidative stress-associated diseases [24].

The purpose of the present study is to investigate the effect of WP on oxidative metabolism in the brain, examine neuronal structure and behavioral changes in diabetic STZ Mice. Our hypothesis is that WP will protect neurons from oxidative stress in the brain which, in turn, may leads to behavioral and morphological improvement in the brain of STZ mice.

## Methods

### Chemicals

Streptozotocin (STZ): (99% pure) and other chemicals were purchased from Sigma chemical Company (St Louis, MO, USA). All other chemicals used were of analytical grade.

### Whey protein extraction

Camel milk was obtained from three breeds (Majaheem, Maghateer and sofr) of camel from the Najd region in Saudi Arabia. The milk was skimmed by centrifugation at 5000 g for 20 min using a IEC Model K centrifuge, [Boston, USA]. Skim milk was acidified to pH 4.3 using 1 M HCl. The precipitated casein was removed by centrifugation, and the supernatant containing the whey protein was brought to 70% saturation with ammonium sulfate and incubated overnight at 4°C. The precipitate containing whey proteins was collected by centrifugation and dialyzed against distilled water for 48 h at 4°C using a Spectra/Pro Membrane, MWCO 6000–8000 Da. The obtained dialyzates were lyophilized using a Unitop 600SL, Virtis Company, Gardiner, New York, USA and were kept at −20°C until used [25].

### Mouse model of diabetes and investigations

This study did not involve endangered or protected animal species. All procedures were conducted in accordance with the standards set forth in the guidelines for the care and use of experimental animals by the Committee for the Purpose of Control and Supervision of Experiments on Animals by the National Institutes of Health, USA. The study protocol (care and handling of experimental animals) was approved by the Animal Ethics Committee of the Zoology Department in the College of Science at King Saud University.

Adult albino male mice weighing 25–30 g, were obtained from the College of Pharmacy, King Saud University, Saudi Arabia and housed in stainless steel wire cages (2 animals/cage) under pathogen-free conditions. The animals were maintained at 18-22°C on a 12:12 h light/dark cycle and provided with food and water *ad libitum*. The diabetic group of animals were intraperitoneal injected with STZ (70 mg/kg) to induce diabetes STZ-injected animals exhibited massive glycosuria and hyperglycemia within 5 days of injection. Diabetes was confirmed in Mice by measuring the fasting blood glucose level (200–250 mg/dl) before use. The animals were assigned into three groups: 1) the first control group (N) was given phosphate buffered saline, 2) the second DM group received STZ (DM), 3) the third DM group was treated orally with whey protein (WP) at a dose of 100 mg/kg/day for 5 days to induce pre-diabetic condition and 21 days for post-diabetic induction.

### Biochemical, behavioral, and histological assays

Eight animals in each group were tested for behavioral changes by using activity cage, rota-rod and grip strength meter. After testing, animals were killed by decapitation. Blood samples were collected for glucose assay, and brains were removed. Brain regions (cerebrum, cerebellum and medulla oblongata) were dissected, immediately cut into small pieces of 3 mm^3^ and fixed in 10% buffered formalin pH 7.4 for 24 h. The tissues were washed with buffered saline to remove excess fixative and then dehydrated in ascending grades of ethyl alcohol 70, 80, 90 and 95% for 45 min each and in two changes of absolute ethyl alcohol for 30 min each. This was followed by two changes of xylene for 30 min each. The tissues were then impregnated with paraplast plus (three changes) at 60°C for 3 h and then embedded in paraplast plus. Sections (4–5 μm) were prepared with a microtome, de-waxed, hydrated and stained in Mayer’s hemalum solution for 3 min. The sections were stained in eosin for 1 min, washed in tap water and dehydrated in ethanol as described earlier. Hematoxylin–eosin-stained sections were prepared [26].

For biochemical studies 0.25 g brain tissue was homogenized in 3 ml of cold saline. The homogenate was centrifuged at 10,000 g for 10 min at 4°C, and the clear supernatant was collected in a microfuge tube (0.5 ml each) and stored at -40C until used.

#### Lipid peroxidation assay

Lipid peroxidation was determined by the reaction with thiobarbituric acid. Malondialdehyde (MDA) formed was determined according to the method of Preuss et al. [27]. Briefly, 1.0 ml brain supernatant was precipitated with 2 ml 7.5% trichloroacetic acid and centrifuged at 1,000 g for 10 min. Clear supernatant was mixed with 1 ml 0.70% thiobarbituric acid, incubated at 80 0C and the absorbance measured at 532 nm. Tetramethoxypropane was used as the standard.

#### DPPH assay

DPPH (2, 2-Diphenyl-1-Picryl Hydrazyl) is relatively stable free radical. The assay was carried out essentially by the method described by Joyeux et al. [28] and modified by Viturro et al. [29]. The bleaching rate of DPPH was monitored at 517 nm in the presence of the sample. In its radical form, DPPH absorbs at 517 nm, but upon reduction by an antioxidant or radical species its absorption decreases. Briefly, tissue hydrolysate (1 ml) was added to a methanolic solution of DPPH (75 μmol L − 1, 4 mL). The mixture was shaken vigorously and left in the dark at room temperature for 60 min, after which the absorbance was measured at 517 nm. The DPPH-scavenging effect (%) was calculated as [(absorbance at 517 control – absorbance at 517 sample)/OD517 control] × 100. α-tocopherol and BHT were used as controls.

#### Cage activity test

The Ugo Basile 47420-Activity Cage was used to record spontaneous co-ordinate activity in mice and variation of this activity in time. This test was performed 3 min for each animals.

#### Rota-rod assay

In this test, a mouse is placed on a horizontally oriented and mechanically rotating rod at 15 rpm. The rod is suspended above a cage floor, which is low enough not to injure the animal, but high enough to induce avoidance of fall. Mice naturally try to stay on the rotating rod, and avoid falling to the ground. The length of time that a given animal stays on this rotating rod is a measure of their balance, coordination, physical condition, and motor-activity [30].

#### Grip-strength meter assay

The Ugo Basile 47200-Grip-Strength Meter suitable for mice automatically measures grip-strength (i.e. peak force and time resistance) of forelimbs in mice. The aim was to assess forelimbs muscle strength. Each animal tested three times and the peak force of each mouse was recorded. The mean of three values for each mouse was recorded.

#### Glucose assay

Blood glucose levels were determined using the Accu-Trend sensor (Roche Biochemicals, Mannheim, Germany).

### Statistical analysis

The Statistical Package for the Social Sciences (SPSS for windows version 11.0; SPSS Inc, Chicago) was used for the statistical analyses. Comparative analyses were conducted by using the general linear models procedure (SPSS, Inc). Also, the data were analyzed using one-way and two-way analysis of variance (ANOVA) followed by LSD computations to compare various groups with each other. Results were expressed as mean ± S.D. The level of significance was expressed as significant at P < 0.05 and highly significant at P < 0.01 [31].

## Results

### Blood glucose level

**Figure 1:**
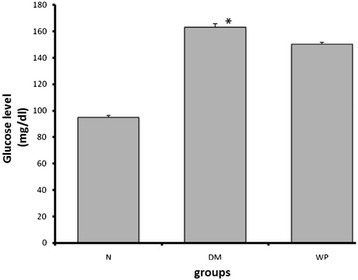
**Shows the blood glucose level in the normal group (N), Diabetic group (DM) and whey protein treated group (WP).** Data are expressed as mean ± SED (N = 6; *p < 0.05, significantly different from the normal group).

The results showed that treatment of animals with WP significantly decreased glucose level in DM mice (Figure 1). The animals of WP group appeared more active and healthy during the behavioral test.

### Behavioral tests

**Figure 2:**
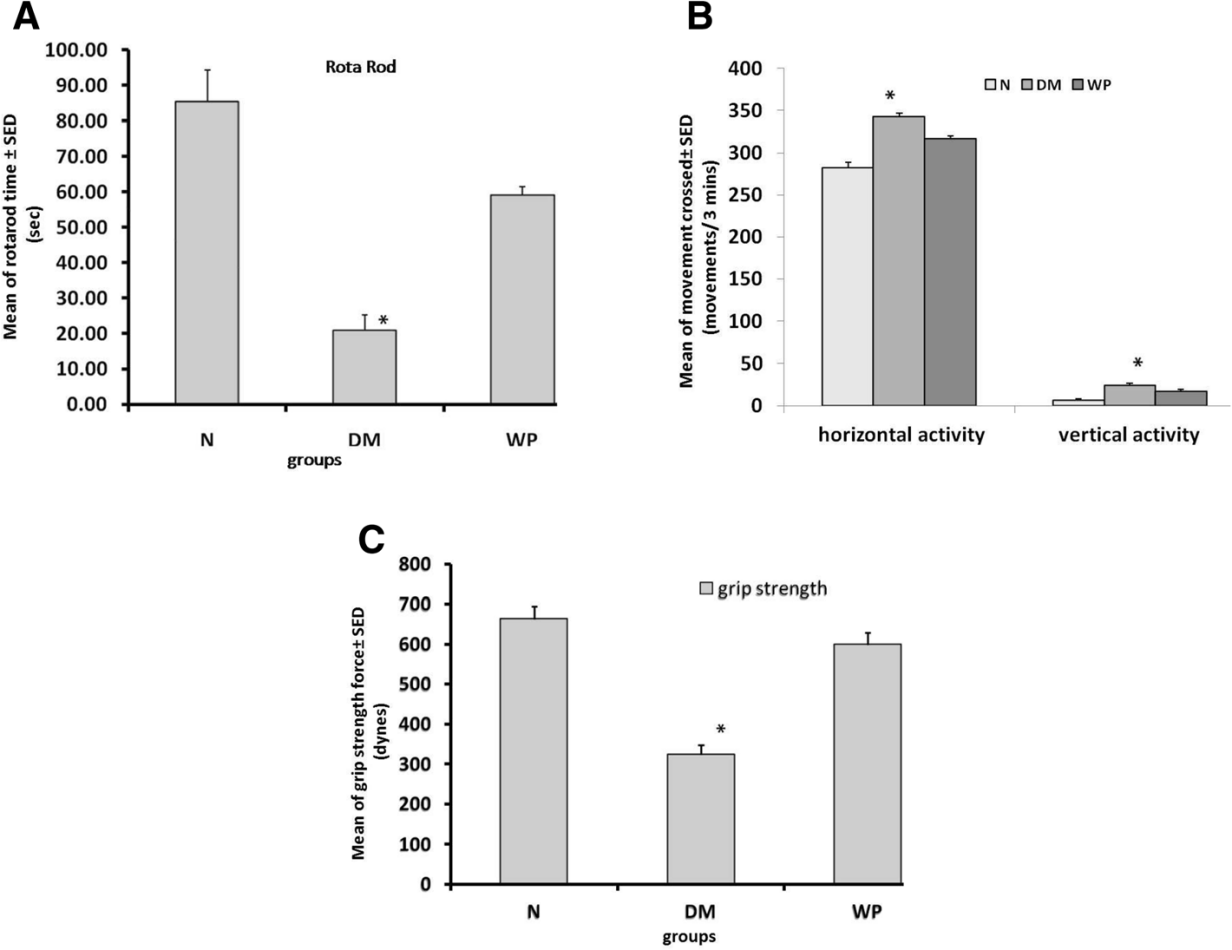
**Shows the rota-rod records. (A)**, vertical and horizontal activities **(B)** and grip strength records for the fore-limb **(C)** in normal group (N), diabetic group (DM) and whey protein treated group (WP). Data are expressed as mean ± S.D. (N = 6; *p < 0.05, significantly different from the normal group).

In rotarod test, the WP treated mice stay on the rotating rod longer than the DM mice (Figure 2A). Treatment of mice with WP improved the balance, coordination, physical condition, and motor activity of the diabetic mice. In the activity cage, the DM group animals appeared anxious and recorded more scores in the horizontal and vertical activities than the normal and WP group of animals (Figure 2B). The WP group of animals showed significant improvements in the grip strength scores and recorded stronger beak than the DM group of animals (Figure 2C).

### Oxidative stress

**Figure 3:**
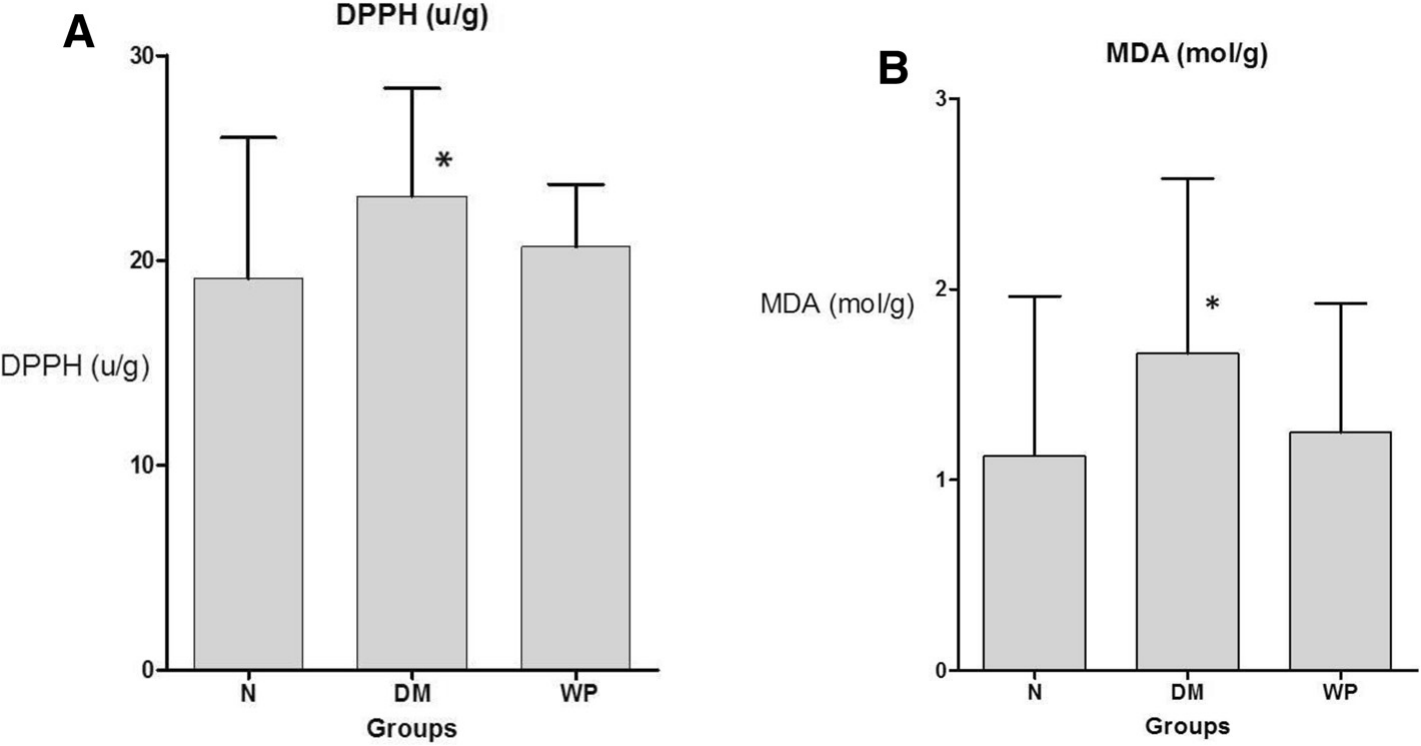
**Shows the DPPH level. (A)** and MAD level **(B)** in the brain tissue of normal group (N), diabetic group (DM) and whey protein treated group (WP). Data are expressed as mean ± SED (N = 6; *p < 0.05, significantly different from the normal group).

DPPH content was significantly increased (P < 0.001) in DM group of animals. In WP-treated animals, the level of DPPH was reduced to the level of DPPH in normal animals (Figure 3A). WP treated mice showed insignificant (P > 0.05) increase in lipid peroxidation whereas a significant increase in MDA (P < 0.001) was seen in DM group (Figure 3B).

### Brain histoarchitecture changes

**Figure 4:**
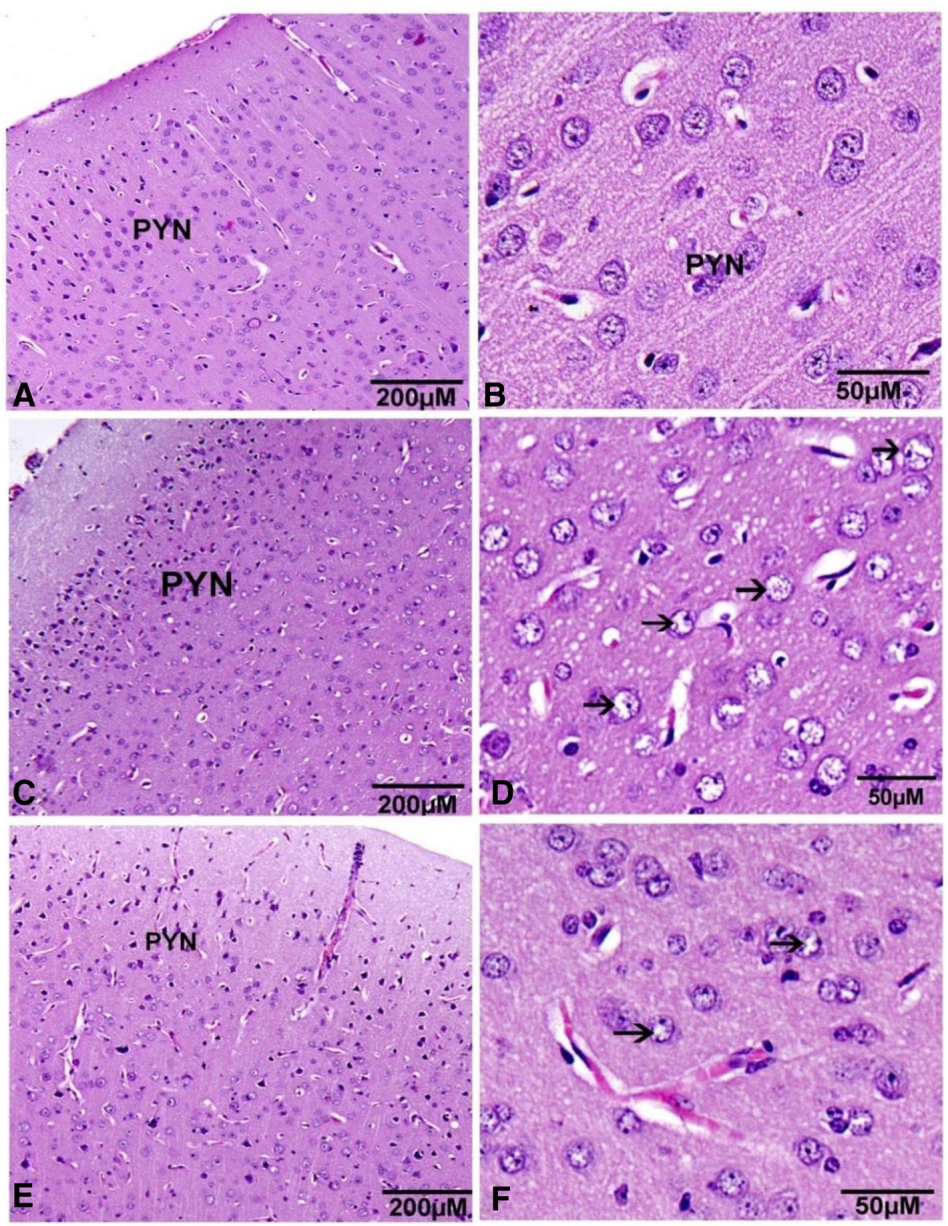
**Sagittal sections in the cerebral cortex show the pyramidal cells distribution (PYN) and neurocyte chromatolysis (arrow). (A, B)** normal group. **(C, D)** diabetic group. **(E, F)** whey protein treated group. (Hematoxylin and eosin stain).

The normal cells of the cerebral cortex had spherical or pyramidal perikaryon whose nuclei were large with neurons arranged in a regular pattern (Figures 4A &B). The cerebral neurons appeared more developed toward the white matter (Figure 4). Pathological changes were observed in many sections in the DM group. Chromatolysis was observed in DM groups and WP treated animals showed significant neuronal protection. (Figures 4D & F).

**Figure 5:**
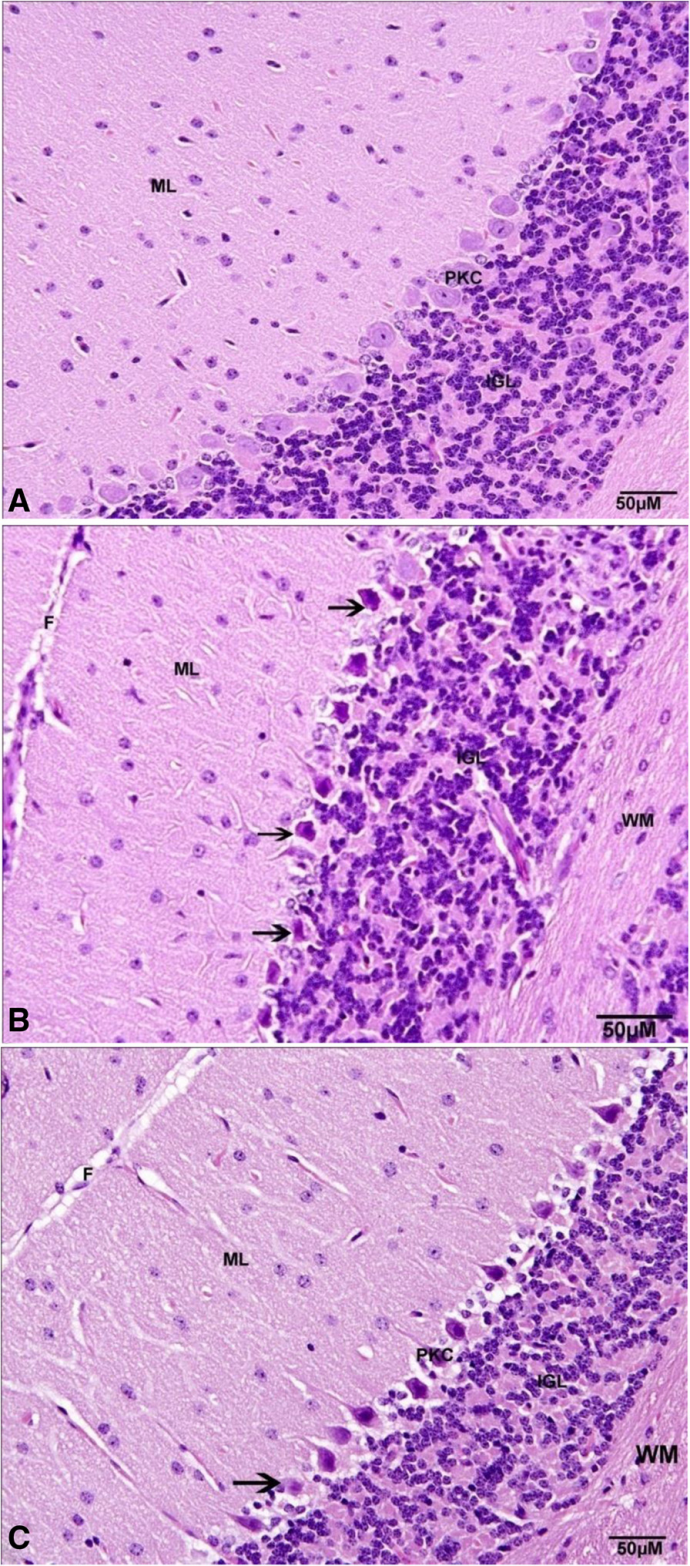
**Photographs of the cerebellar cortex show the degenerated Purkinje cell (arrow), fissure (F), internal granular layer (IGL), molecular layer (ML), Purkinje cell (PKC), Purkinje cell layer (PCL), and white matter (WM). (A)** normal group, **(B)** diabetic group, **(C)** whey protein treated group. (Hematoxylin and eosin stain).

**Figure 6:**
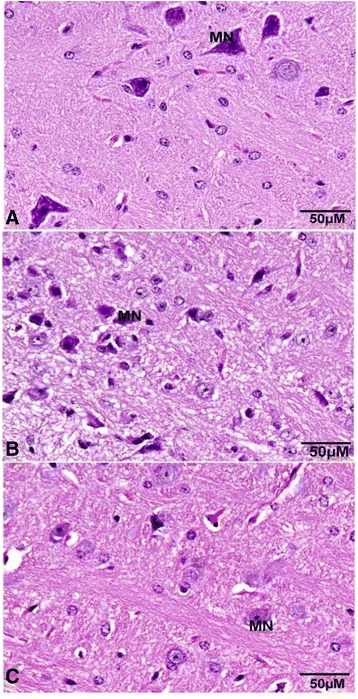
**Sagittal sections in the medulla oblongata show the medulla neurons (MN). (A)** normal group, **(B)** diabetic group, **(C)** WP group. (Hematoxylin and eosin stain).

In the cerebellum, the numbers of neurons in the molecular layer of control mice (Figure 5A) were the higher compared to diabetic and WP group of animals. The control Purkinjee (PKC) cells were arranged in a single row of large neurons with pear-shaped perikaryon and large nucleus (Figure 5A). The lateral processes disappeared and the apical processes formed the permanent dendritic tree (Figure 5). In DM group (Figure 5B), some degenerated and pyknotic Purkinje cells were detected and some were more spindle-shaped and small (Figure 5C). The normal medulla neurons appeared large in size, varied in shape and had round nuclei (Figure 6A). In DM group, most of medulla neurons appeared small and pyknotic (Figure 6B). WP group medulla neurons showed improvement (Figure 6C).

## Discussion

The results of this study demonstrate that hyperglycemia causes abnormalities in the neurobehavior of DM group animals such as physical balance, coordination and grip strength. Biochemical studies showed that DM is associated with disturbance in oxidative stress and neuronal pathology. Neuronal death may lead to cognitive deficits and an increased risk of brain complications [32]. In the diabetic animals, several brain alterations have been described, such as increased lipid peroxidation and DPPH radicals, neuronal changes in the cerebrum, cerebellum and medulla oblongata. Recently, a significant body of evidence to indicate that diabetes has detrimental effects on brain functions such as memory loss in type I and type II diabetes [33]. Some investigators have also reported a reduction in the length of the dendritic trees of the Purkinjee cells and pyramidal cells in diabetic rodents [34]. The diabetic animals show changes in dendritic morphology, probably associated with synaptic disturbances. This may explain memory and learning deficits [3]. Oxidative stress is widely accepted as playing a key mediatory role in the development and progression of diabetes and its complications, due to the increased production of free radicals and impaired antioxidant defenses [35]. Several mechanisms can contribute to increased oxidative stress in diabetic patients, especially chronic exposure to hyperglycemia. Accumulated evidence points out that hyperglycemia can lead to elevated ROS and reactive nitrogen species (RNS) production by the mitochondrial respiratory system [36], glucose autoxidation [37], activation of the polyol pathway [38], formation of advanced glycation end products [39], antioxidant enzyme inactivation and an imbalance of glutathione redox status [40]. Hyperglycemia can promote an important oxidative imbalance, favoring the production of free radicals and the reduction of antioxidant defenses. At high concentrations, ROS/RNS can damage the major components of the cellular structure, including nucleic acids, proteins, amino acids, and lipids [41]. Such oxidative modifications in the diabetes condition would affect several cell functions, metabolism, and gene expression, which in turn can cause other pathological conditions [42]. The oxidative stress leads to neuronal damage in several brain regions [5]. For example, neuronal loss in cerebrum impairs animal’s memory [43], neuronal loss in cerebellum can have effect on balance and coordination [6] and neuronal loss in medulla oblongata and spinal cord can affect physical activity of mice [44].

Supplementation with WP for 26 days decreased blood glucose and showed significant improvement in the physical balance, coordination, motor activities, and muscles strength in diabetic animals. WP supplement also decreased lipid peroxidation and DPPH radicals. Overall, this study demonstrated that WP supplementation significantly improved pathological alterations in diabetic mice as reported by Ebaid et al. [25]. WP has been found to significantly suppress hydroperoxide and ROS levels in liver and other tissues in mice by stimulating production of glutathione synthesis and thereby boosting cellular antioxidant defense [23]. Therefore, we suggest that WP may be an important therapeutic tool to combat oxidative stress-associated diseases [24]. We propose that WP may ameliorate diabetes in DM mice by its ability to neutralize free radicals and thereby prevent neuronal damage caused by oxidative stress.

## Conclusions

WP has a unique protective effect on glucose metabolism in STZ diabetic mice. WP supplementation improves the behavior of diabetic mice and reduces neuronal damage in the brain caused by oxidative stress.
